# Cognitive behaviour therapy plus aerobic exercise training to increase activity in patients with myotonic dystrophy type 1 (DM1) compared to usual care (OPTIMISTIC): study protocol for randomised controlled trial

**DOI:** 10.1186/s13063-015-0737-7

**Published:** 2015-05-23

**Authors:** Baziel van Engelen, The OPTIMISTIC Consortium

**Affiliations:** Neurologist, Department of Neurology, Radboud University Medical Centre, Reinier Postlaan 4 (route 935), PO Box 9101, 6500 Nijmegen, The Netherlands

**Keywords:** Myotonic dystrophy type 1, DM1, Cognitive behavioural therapy, Aerobic exercise training, Rare diseases

## Abstract

**Background:**

Myotonic dystrophy type 1 (DM1) is a rare, inherited chronic progressive disease as well as an autosomal dominant multi-systemic disorder. It is probably one of the most common adult forms of muscular dystrophy, with a prevalence of approximately 10 per 100,000 people affected. With 733 million people in Europe, we estimate that 75,000 people in Europe are affected with DM1.

**Methods/Design:**

OPTIMISTIC is a multi-centre, randomised trial designed to compare an intervention comprising cognitive behavioural therapy (CBT) plus graded exercise therapy against standard care. Participants will be recruited from myotonic dystrophy clinics and neuromuscular centres in France, Germany, the Netherlands and the United Kingdom. A sample size of 208 individuals is needed. To allow for some potential loss to follow-up, a total of 296 male and female patients aged 18 years and older with genetically proven classical or adult DM1 and suffering from severe fatigue (only DM1 patients with a Checklist Individual Strength (CIS) subscale fatigue severity score ≥35 are likely to benefit from the intervention), able to walk independently and able to complete the trial interventions will be included.

The primary outcome of the study is the score on the DM1-Activ scale, which is a measure of activity and participation for patients with DM1. Secondary outcomes include the 6-minute walk test, objective physical activity measured with an accelerometer, quality of life and cognitive measures. The trial will also collect data on potential effect modifiers of the short- and long-term clinical response, including pain, muscular impairment and cognitive-behavioural variables. In addition, OPTIMISTIC will identify genetic factors that predict outcome and potential biomarkers as surrogate outcome measures that best explain the observed clinical variation.

**Discussion:**

OPTIMISTIC will not only provide effectiveness data on an intervention that could fill a treatment-gap for DM1 patients but will also improve our understanding of the relevant determinants of the prognosis of DM1.

**Trial registration:**

Registration number: Cinicaltrials.gov NCT02118779; registered 11 April 2014.

## Background

Myotonic dystrophy type 1 (DM1) is a rare, inherited, progressive disease as well as an autosomal dominant multi-systemic disorder. It is one of the most common adult forms of muscular dystrophy, with a prevalence of approximately 10 per 100,000 people affected [1, 2]. With 733 million people in Europe, we estimate that 75,000 are DM1 patients [3]. Typical symptoms of the disease include progressive muscle weakness and wasting from distal to proximal, ptosis; weakness of facial, jaw and anterior neck muscles; myotonia; daytime sleepiness; fatigue and cataracts. Other symptoms of adult DM1 include cardiac conduction defects, as well as endocrine, gastrointestinal and cognitive dysfunction. DM1 is one of the most variable human diseases, has complex, multi-systemic and progressively worsening clinical manifestations and leads to severe physical impairment, restricted social participation and premature death [4, 5].

There is no pharmaceutical treatment for causal or symptomatic relief of DM1 core symptoms, with the exception of Modafinil for excessive daytime sleepiness and mexiletine for myotonia. Thus, the aim of treatment is to relieve impairments, reduce limitations and optimise participation. Physical activity has been acknowledged as an important factor for health in general. For patients with a slowly progressive neuromuscular disease, such as DM1, there is accumulating evidence for prescribing low-to-moderate-intensity strength and aerobic exercise training, and an active lifestyle [6]. Nevertheless, recent reviews conclude that existing studies are limited in number and quality, and that there is a need for disease-specific, randomised, controlled trials investigating the effect on quality of life [6–8].

### Rationale for the study

It was demonstrated recently by OPTIMISTIC Partner 1 (see *The OPTIMISTIC Consortium* for list of partners) that severe fatigue, defined as a score equal to or higher than 35 on the subscale fatigue of the Checklist Individual Strength (CIS-fatigue), was reported by around 70 % of patients with DM1 [9]. These severely fatigued patients had more problems with physical and social functioning as well as with their mental and general health than similar patients without severe fatigue. They also had more problems with concentration and planning. As such, experienced fatigue should be clearly distinguished from muscle weakness, which is probably the most common and characteristic symptom of DM1 and also of a lack of initiative (apathy) that is known to occur often in DM1.

**Fig. 1:**
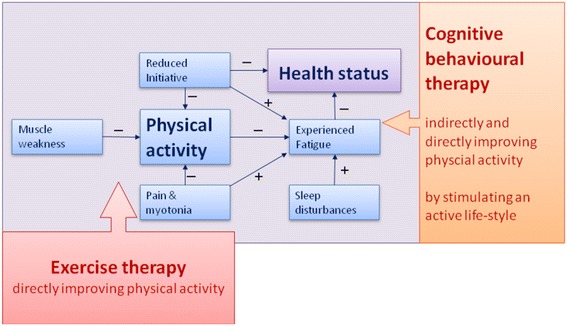
Determinants of health status in DM1. Exercise therapy is intended to improve physical activity whereas cognitive behavioural therapy is intended to stimulate an active life-style by addressing perpetuating factors of fatigue such as lack of physical activity, motivation, pain and sleep disturbances and also by teaching patients to compensate for a lack of initiative (motivation)

In a longitudinal study, we built a model of perpetuating factors for fatigue in patients with DM1 (Fig. 1). It appeared that lack of physical activity, sleep disturbances and pain all contributed to experienced fatigue. In addition, loss of muscle strength and pain contributed to fatigue through a lower level of physical activity. Ultimately, experienced fatigue and physical activity both contributed to the level of societal participation [10]. A lack of initiative (motivation in the model of Fig. 1) further increased fatigue but also had a direct negative effect on societal participation. Thus, theoretically, in order to improve societal participation one should compensate for a reduced initiative, optimise physical activity and alleviate experienced fatigue. To alleviate fatigue one should address the fatigue maintaining factors identified by the model, for example, experience of pain or sleep disorders.

The main rationale for the combination of CBT and physical exercise training is based on our DM1-specific model [10] (Fig. 1). The DM1-specific model shows that physical activity, experienced fatigue and lack of initiative are the main determinants of DM1 health status. OPTIMISTIC is the first model-based clinical trial in DM1. It evaluates the effect, and the maintenance of effects, of CBT combined with exercise training on the reduction of chronic fatigue in patients with DM1.

Importantly, the intervention will also involve caregivers where they are willing to take part. The disabilities associated with DM1 put considerable strain on caregivers and can also lead to a negative interaction with the patient. The intervention will aim to support caregivers by installing realistic expectations about what can be expected from the patient, teach caregivers how to help patients to stay as self-reliant as possible and also reduce caregivers strain by taking time for themselves. If a DM1 patient has no caregiver or significant other, or no caregiver or significant other willing to take part in the study, the patient will not be excluded from the study. All patients will be asked if the study team can approach him or her to inform them of further research. This contact does not constitute consent.

### Objectives

#### Primary objective

The primary objectives of this study are to evaluate the effect of a tailored behavioural change intervention comprising CBT and graded exercise on participation (as measured by the DM1-Activ scale) for severely fatigued patients with myotonic dystrophy type 1 compared to standard care.

#### Secondary objectives

The secondary objectives of this study are as follows:

1. the creation and introduction of evidence based clinical guidelines on exercise and cognitive behavioural therapy combined with graded exercise in DM1,
2. the identification of individual (serum, deoxyribonucleic acid (DNA)) or composite biomarker profiles as surrogate outcome measures and moderating or mediating factors of the efficacy and safety of the clinical response, and
3. the creation of a clinical trial infrastructure for European DM1 trials, including the collection of natural history data from a large cohort of DM1 patients.

## Methods/Design

### Study description

OPTIMISTIC is a two-arm, multi-centre, randomised controlled trial designed to compare cognitive behavioural therapy, plus graded exercise against standard patient management regimes. It is expected that the trial and outcome work will lead to new clinical guidelines for DM1 management. The intervention comprises cognitive behavioural therapy (CBT) and graded exercise therapy, both of which aim to achieve a more active lifestyle.

**Fig. 2:**
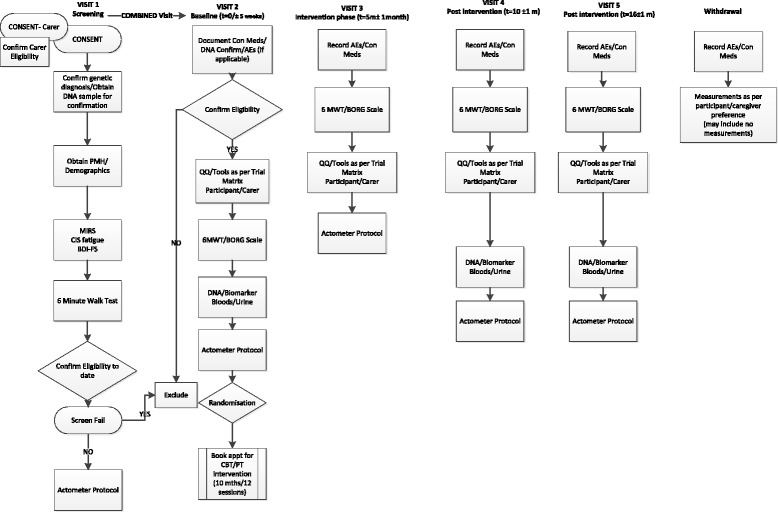
OPTIMISTIC patient pathway. Visit 1 and visit 2 can be combined if appropriate and acceptable to participant. Duplicate tests and questionnaires will not be performed

The effectiveness of this intervention, together with any adverse events associated with it, will be compared to standard patient management. Outcome measures will be measured at baseline; 0 to 5 week period depending if separate screening and baseline visits are required, 5 months (± 1 month, 10 months (± 1 month, the end of the intervention period) and at 6 months (± 1 month) post intervention (that is, 16 months from baseline, Fig. 2).

The baseline period constitutes either a combined screening/baseline visit if the genetic diagnosis of DM1 is confirmed or a separate screening visit, in which to obtain a blood sample for DM1 genetic status, followed by a baseline visit, if confirmed. Depending upon the medical and DM symptom history obtained from non-confirmed participants, they may carry out all screening and baseline activities while awaiting the genetic confirmation. This will allow those participants to enrol into the screening phase of the study prior to genetic confirmation. It is of the opinion of the specialists that non-confirmation will occur in very few cases.

Participants will not be blind to allocation, but the study outcome measures listed in the Appendix will be collected by staff who are blind to allocation.

### Study population

#### Number of participants

Participants will be recruited from myotonic dystrophy clinics and neuromuscular centres in the UK, the Netherlands, Germany and France. A total of 296 (male and female) patients will be recruited (see *Inclusion criteria* and *Exclusion criteria*). The figure of 296 allows for drop-outs; 208 are needed to complete the study with regard to our primary outcome (the DM1-Activ scale). This sample size for the primary outcome means that the study is also powered for one of the secondary outcomes, the 6-minute walk test. See *Sample Size Calculation* for more details with regard to participant numbers.

**Fig. 3:**
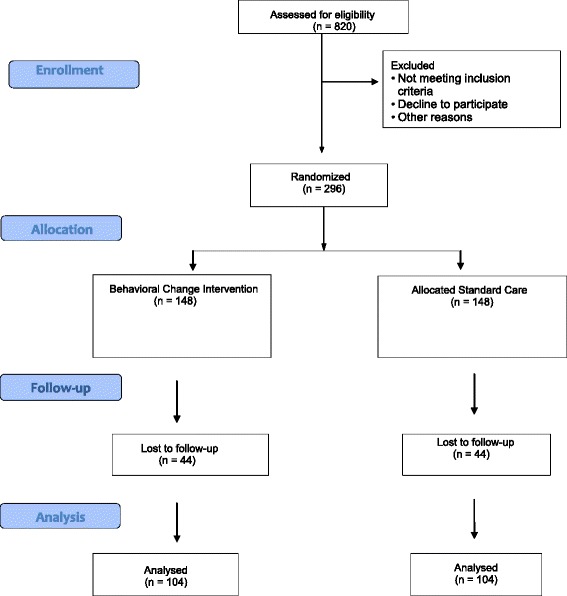
OPTIMISTIC Study flowchart

Participants will be randomised to receive a 10-month tailored behavioural change intervention (n = 148) or standard care (n = 148). The flow of participants through the trial is shown in Fig. 3.

For the biomarker study and DNA analysis, whole blood will be collected in a standardised manner from all 296 DM1 participants enrolled in the trial, with 3 × 10 ml whole blood samples collected per patient and sent to a central sample storage facility, the University of Newcastle Biobank. This facility will undertake DNA and serum preparation and send one whole blood sample per participant to Nijmegen University for ribonucleic acid (RNA) preparation.

Samples will be collected at baseline, 10 months (the end of the intervention period) and at 6-month post-intervention (that is, 16 months from baseline). All samples will be obtained, processed and stored as per the Study Operations Manual, which addresses local policies and guidelines.

#### Inclusion criteria

Judgements as to whether a potential participant meets an inclusion criterion are made by the treating clinician unless stated otherwise. Inclusion criteria are as stated below:

1. Able to provide informed consent.
2. Genetically proven DM1, aged 18 years and older, suffering from severe fatigue (CIS-fatigue severity subscale score ≥35) [11]. The genetic diagnosis and level of fatigue will be determined as part of the eligibility screening process.
3. Ability to walk independently (ankle-foot orthoses and canes are accepted).

Carers inclusion criteria involve the ability to give informed consent and to complete study questionnaires.

#### Exclusion criteria

Judgements as to whether a potential participant meets an exclusion criterion are made by the treating clinician unless stated otherwise. Exclusion criteria are as stated below:

1. Neurological or orthopaedic co-morbidity interfering with the interventions or possibly influencing outcomes.
2. Use of psychotropic drugs (except Modafinil, Ritalin and antidepressants where the dosing regimen has been stable for at least 12 months prior to screening). If the doses of Modafinil or Ritalin increase during the 10 months of intervention/non-intervention then the participant will be excluded.
3. Severe depression at screening as per clinical judgement.
4. Participation in another clinical trial of an investigational medicinal product (CTIMP) or other interventional study considered to influence outcomes being evaluated in OPTIMISTIC concurrently or within 30 days prior to screening for entry into this study.
5. Unable to complete study questionnaires.

The exclusion criteria for carers are the inability to give informed consent, complete questionnaires and attend CBT sessions with participants.

#### Participant selection and enrolment

##### Identifying participants

The study will be conducted on an outpatient basis. The primary route of participant recruitment will be through their neurologist at myotonic dystrophy clinics and neuromuscular centres in the UK, the Netherlands, Germany and France. At each centre, the PI and neurological physicians will be assisted by the study research nurses who will identify potential participants by assessing inclusion and exclusion criteria.

Additionally, participants will also be recruited from the local or national registries of individuals with DM1. Currently, the registries for France, Germany, the Netherlands and the UK contain 1,500, 500, 400 and 300 registered individuals, respectively. Registries contain patients who have specifically shown interest in participating in research and previously consented to be contacted about research in which they can take part. Registries will contact all potentially eligible participants providing a Patient Information Sheet (PIS) and local contact details as well as contacting medical professionals who have shown an interest in the condition to increase awareness beyond the trial sites.

As DM1 is a genetic condition, it is likely that some members of the same family will wish to participate in the study. Immediate family members (mother, father, children, and siblings) may request to attend together for screening and subsequent study visits or separately at a later date. There is no time limit on when a subsequent family member can attend for screening. To avoid treatment contamination, immediate family members will be allocated to the same group, with the first family member being randomly allocated and subsequent member(s) being assigned to the same group as the first. The web-based Tayside Randomisation SysTem (TRuST) will assign the initial family member as per stratification and minimisation criteria (see *Randomisation*), with subsequent family member(s) being assigned by TRuST into the same group. All participants will be asked for consent to allow the study team to enquire if another member of their immediate family is enrolled into OPTIMISTIC. Their name will be required to review the randomisation log to establish their study ID. The study ID of the initial family member will be entered into TRuST, thereby allowing same group allocation.

It may be the case that the carer looks after more than one member of the family wishing to take part. They will be given the opportunity to participate for all family members. However, the carer can opt to not participate at all or to take part for one or all of the family members, whatever is convenient for them.

##### Communication and dissemination

A communication strategy will be implemented involving patient organisations and patients from across Europe in order to inform and engage the wider myotonic dystrophy community. Information will be provided for patient organisations to disseminate through newsletters, websites and at conferences where appropriate. OPTIMISTIC also has links to the global neuromuscular network TREAT-NMD (www.treat-nmd.eu) and the progress of the study will be announced through their established newsletter (circulation over 3,000) allowing communication of the study to medical professionals and experts in the field at an international level.

A dedicated project website (www.optimistic-dm.eu) will provide local site information and provide contact details for patients while providing background information about the project to all interested parties. A dedicated project newsletter will be circulated to all participants.

Lay-friendly results will be disseminated to participants and through patient organisations, the TREAT-NMD website and the OPTIMISTIC project website.

##### Consenting participants

Potential participants recruited via myotonic dystrophy clinics will receive a Patient Information Sheet (PIS) from their physician, research nurse or suitably qualified member of the study team and will have the opportunity to discuss the study in detail with study staff. With a patient’s permission, caregivers and immediate family members will also be invited to participate (see *Interventions*). Information Sheets and consent forms have been reviewed and contributed to by patient representatives and patient organisations.

Potentially suitable participants registered with local research networks and who have consented to being contacted about research studies will be approached by the study research nurse using the potential participant’s preferred method of contact. Patients identified via local patient registers will be sent Information Sheets: Brief Patient Information Sheet and the full Patient and Carer Information Sheets and will be asked to call or email if they wish to participate in the study. The research nurse or a member of the study team will then contact the individual and answer any questions he or she may have about the study. A contact telephone number and e-mail address of a member of the local study team will be included on the PIS to give the participant the opportunity to discuss the study prior to their clinic visit, if appropriate.

Those participants who have received the PIS in the post will be asked to call/email or return the reply slip if they wish to participate in the study. A log will be kept by the research nurses or a member of the study team of all participants invited to participate. For those participants who received the PIS prior to their routine clinic appointment and had the opportunity to ask questions and wish to proceed to screening on that day, informed consent will be obtained and, if appropriate and convenient for the patient, they will then proceed to study procedures as outlined in Fig. 2 and Appendix. Alternatively, a convenient appointment will be made for another date. A patient specific section of the study website (www.optimistic-dm.eu) containing PISs and research staff contact details will be available in the various languages. The research nurse or a member of the study team will contact participants who have been sent the PIS after 10 days if they have not responded to make sure they had received the PIS and do not wish take part in the study.

Participants approached at a clinic, or who have received study information from their neurologist and have indicated interest by returning the reply slip, e-mailing or calling the study team will be invited to attend a study screening or combined screening/baseline visit if appropriate. All participants will be given at least 24 h to consider their response. Informed consent will be obtained by the PI, the research nurse or a suitably qualified and trained member of the study team as recorded on the Site Delegation of Responsibilities Log. We will obtain informed consent from all participants taking part in OPTIMISTIC.

All individuals taking informed consent will have received training in Good Clinical Practice (GCP) and trial information and procedures specific to OPTIMISTIC. It will be explained to patients that they are under no obligation to enter the trial and that they can withdraw at any time during the trial without having to give a reason. A copy of the signed participant consent form, along with a copy of the PIS, will be given to the study participant. The original signed consent form will be retained at the study site and filed in the Investigator Site File (ISF), with a copy in the medical notes. The participant’s general practitioner (GP) will be informed of their study participation as per clinical and national requirements. Consenting carers will receive a copy of their consent form and PIS with the original being filed in the ISF.

If new safety information linked to the interventions being evaluated results in significant changes to the study risk-benefit assessment, the Protocol, participant PIS and/or consent form will be reviewed updated and amended as necessary. All participants, including those already being treated, will be informed of the new information, given a copy of the revised consent form and asked to re-consent if they choose to continue in the study.

Participants and carers will be invited to complete a series of questionnaires at each study visit. Questionnaires should be attempted but due to patient fatigue portfolio a priority list will be developed and those not completed will be documented as missing data and is reflective of carrying out a study in a fatigued population (Fig. 2). In addition, all study assessments not completed will be documented as missing data.

##### Ineligible and non-recruited participants

The reason(s) for ineligibility will be explained to all individuals who are clearly not eligible, or who provide consent but then fail screening. Any questions they have will be answered. They will be thanked for their participation in screening and any relevant clinical information will be added to their hospital notes. The study team will document the patients past medical history and specific DM1 disease progression history. For some patients this may be an extensive report; therefore, the researcher will obtain the information at screening and/or at baseline visit if required.

### Outcomes

See Appendix for the trial outcome measurement schedule.

#### Primary outcome

The primary outcome measure will be the DM1-Activ measured at the end of the 10-month intervention period. DM1-Activ is a specific outcome measure of activity and participation for patients with DM1 [12].

#### Secondary outcomes

Secondary outcomes are categorised into five groups:

##### 1. Activity (measurement time 50 min)

- 6-minute walk test (6MWT) with BORG Scale assessment (0 to 10 rating of perceived exertion score)
- Activities of Daily Living (ADL) assessment
- Myotonic Dystrophy Health Index (MDHI)
- Physical activity measured with an actometer (NB: participants take this home after each visit and wear for 2 weeks).

##### 2. Fatigue and sleepiness (10 min)

- Fatigue and Daytime Sleepiness Scale (FDSS)
- Checklist Individual Strength (CIS) subscale fatigue severity

##### 3. Quality of life (20 min)

- Individualised Neuromuscular Quality of Life Questionnaire (InQoL)

##### 4. Mood (15 min)

- Beck Depression Inventory Fast Screen (BDI-FS)

##### 5. Cognitive (20 min)

- Apathy evaluation scale (AES)
- Stroop colour word test

#### Measures used as potential effect modifiers

We will collect some data to evaluate their potential as modifiers of the effect seen in the trial:

1. Muscular impairment rating scale (MIRS);
2. McGill pain questionnaire;
3. Cognitive behavioural variables (self-efficacy scale for fatigue (SES-28), Jacobsen Fatigue catastrophising scale (FCS), Focusing on symptoms (IMmQ), Illness acceptance scale, Social support list discrepancy (SSL-D)/Interactions (SSL-I)/Negative Interactions (SSL-N);
4. Trail making test; and
5. Adult Social Behaviour Questionnaire (ASBQ).

### Identification of biomarkers and expansion of the dystrophia myotonica-protein kinase cytosine, thymine, guanine (CTG) repeats

Whole blood will be collected in a standardised manner from all 296 DM1 participants enrolled in the trial, with 3 × 10 ml whole blood samples and up to 20 ml urine sample collected per patient. These will be used for biomarker identification and for genetic work linked to the dystrophia myotonica-protein kinase (DMPK) CTG repeats.

#### Identification of biomarkers

mRNA and microRNA expression changes in serum samples will be collected for each participant in the trial (that is, up to 296 DM1 patients). In particular, attention will be paid to the expression of candidate microRNAs relevant to the following targets in addition to any others selected from initial next generation RNA sequencing studies in the discovery cohorts:

1. insulin receptor,
2. muscle chloride channel,
3. SERCA1,
4. RyR1 and
5. troponin T.

The following microRNA targets will be assessed in addition to any further selections based on other sources:

1. miR-1,
2. miR-133b,
3. miR-29 and
4. miR-206.

#### Expansion of CTG repeats

The blood samples collected for DNA extraction will also be collected at the baseline and at the end of the observation period (that is, 16 months after the start of the intervention or control). Also, historical DNA samples will be collected, if available, to inform analysis of disease progression. DM1 is caused by the expansion of CTG repeats in the *DMPK* gene. Disease severity is correlated with the number of repeats. However, the CTG repeats are highly unstable and the number of repeats changes from one generation to the next and throughout life. Recent data have indicated that the major modifier of disease severity is the number of CTG repeats inherited and that disease severity is further modified by the individual specific rate of somatic expansion [13, 14]. The time series of DNA samples collected will be used to estimate the number of repeats inherited and monitor the rate of change of the repeat length over time in each patient.

### Cardiac magnetic resonance image sub-study

Cardiac magnetic resonance imaging (CMRI) uses a combination of harmless radiofrequency (RF) waves and powerful magnets to cause hydrogen nuclei within the cardiac cell molecules to vibrate and emit RF energy. The MRI scanner detects the energy emissions and converts them to viewable images. When diseases begin, there are changes in the heart’s tissue. Because even minor changes in tissue affect the rates at which energy is emitted, many medical conditions can be detected at their very early stages. MRI is safe; there is no ionizing radiation, and the contrast agents are non-nephrotoxic and non-allergenic. Until recently, the use of MRI in detecting heart disease was limited due to the technical challenges of imaging a moving object. Hardware advances and improvement in pulse sequence design now permit high resolution imaging of the beating heart, allowing physicians to view cardiac motion in a way not previously possible. MRI is done to evaluate the structure and function of the heart and blood vessels. MRI may provide information that cannot be obtained by other tests such as chest X-ray, electrocardiography (ECG), echocardiogram, or nuclear tests.

The study CMRIs will be performed at the Newcastle Centre (NMRC) at baseline and repeated at the end of the intervention period. The CMRI examinations will be performed with the contract enhancement gadolinium, in 40 eligible participants, with 20 in each group. Participants will undergo:

1. cardiac cine imaging, to evaluate cardiac morphology;
2. systolic and diastolic function; and
3. cardiac tagging to evaluate wall motion and torsion.

These measurements will be taken with the participants lying supine within a Philips 3T MRI scanner. After a short break, participants will lie in the scanner prone for phosphorus magnetic resonance spectroscopy, to measure the ratio of polymerase chain reaction (PCr)/adenosine triphosphate (ATP), a measure of steady state metabolite use.

MRI safety will be established prior to baseline and end of intervention scan. This includes; assessing for *in vivo* ferrous material, claustrophobia, abnormal renal function and pregnancy. The PI will assess renal function from the participant’s medical notes at screening, if required a blood tests for urea and electrolytes (U&Es) will be requested. In addition, child-bearing potential female participants will consent to have a urine pregnancy test performed.

Participants that have had a baseline CMRI and withdraw from the study prior to the end of the intervention period, will be invited to have an end of study CMRI if the period from their initial CMRI is greater than 3 months. No further statistical review is required for the CMRI sub-study as only frequencies and associations will be assessed.

### Magnetic resonance muscle (MRmuscle) sub-study

Magnetic resonance (MR) biomarkers that have been found to be extremely useful in the evaluation and understanding of other muscular diseases (that is, facioscapulohumeral muscular dystrophy (FSHD)) are the fractional muscle and fat levels in muscles and the presence of inflammation [15]. It was observed that the fat infiltration measured in this way already occurs before clinical symptoms or decline in muscle force is noticed. Moreover, fat infiltration is unevenly distributed both intra- and intermuscular and from the time dependence of this pattern a characteristic progression of the disease was established that could be related to the origin of the disease.

This sub-study will perform MR in the upper and lower legs of DM1 patients to establish the value of fat infiltration as a biomarker of the disease. In addition, the presence of oedema will be estimated using the so called, turbo inversion recovery magnitude (TIRM) sequence as a biomarker of inflammation. These biomarkers will be followed quantitatively during the treatment intervention. MRmuscle is a non-contrast MR.

The overall aim of this MRmuscle sub-study is to obtain MR biomarkers that can be used as clinical endpoints for therapeutic interventions in patients with myotonic dystrophy. This sub-study will only be performed in two of the participating sites; Radboud University Medical Centre, Nijmegen and Centre hospitalier universitaire Henri-Mondor, Paris. Based on previous findings in FSHD, the desired number of participants will be at least 30 (15 from the intervention group and 15 from the comparison group). Taking into account potential loss to follow-up, the study will aim to recruit up to 50 patients to meet the target of 30 completed sets of MR data. The number of participants per centre may not be equally divided (for example, Nijmegen 20 and Paris 30 participants.) This multi-centre design increases the credibility of the MRdata. Each participating centre will employ its own quality assurance program/procedure. This procedure is inherit within each MR department and usually allows the study MR protocol to be carried out initially on a normal volunteer to set baseline parameters.

Participants that are eligible for the OPTIMISTIC main study and have no contraindications for MRI can be included in the MRmuscle sub-study. The MRmuscle sub-study will stop recruiting once 50 participants have been recruited, or the OPTIMISTIC main study stops recruiting, whichever comes first.

MR contraindications will be as per local guidelines but will include having metal parts in the body, having a pacemaker, claustrophobia, being unable or unwilling to tolerate the loud noises made by the MR machine and a body mass index (BMI) that is contraindicated for fitting comfortably into the MR scanner.

Patients will receive information for MRI together with the main OPTIMISTIC participant information. The MR informed consent form will be signed at the same time as the main OPTIMISTIC informed consent form. It will be possible for a participant to consent to the main OPTIMISTIC study but not the MRmuscle sub-study.

MR measurements will be performed on a 3T MR system (TIM Trio, Siemens, Erlangen, Germany). Patients will undergo an MR for the upper and lower legs. Prior to MR imagining a fish oil capsule will be positioned at one third of the distance between the spina iliaca anterior superior and the patella and will serve as a landmark for exact matching of the imaging slices between the baseline and follow-up measurement. The total time of the measurement is approximately 30 min. Measurements will be performed during the baseline period, before the first CBT session for those randomised into the intervention group and repeated at the end of the intervention period for all participants. For participants that have had a baseline MR and withdraw from the study prior to the end of the intervention period, these participants will be invited to have an end of study MR if the period from their initial MR is greater than 3 months.

Baseline biomarker values will be collected for the following:

1. cross sectional area of muscle,
2. muscle/fat quantification, and
3. signal intensity per region of interest (ROI) oedema.

This data will be used to:

1. investigate whether there is a relation with the state of the disease and
2. investigate whether these values change due to the intervention, that is, comparison with patients in the control group.

Differences between affected and non-affected muscles will be correlated with each other and with the values of various parameters determined in OPTIMISTIC (for example, muscle strength). Specific biomarkers established for DM1 will then be used to evaluate the effect of the CBT intervention.

The acquired MR images will be anonymised and will only contain participant’s study ID.

In Nijmegen, the anonymised data will be stored on a hospital server. In Paris, the anonymised data will copied onto an encrypted CD or DVD and sent by courier to Nijmegen where the data will be uploaded onto the server for analysis by the MR research team.

### Randomisation

Randomisation will be via a centrally controlled web-based, GCP-compliant randomisation system, run by Tayside Clinical Trials Unit (TCTU) called TRuST (Tayside Randomisation SysTem).

Randomisation will occur as indicated below:

1. It will be stratified by site.
2. It will be minimised for baseline DM1 severity measured by the Muscular Impairment Rate Scale (MIRS) 5-point scale.
3. It will be minimised for baseline involvement (or not) of a caregiver.

For immediate family member participation TRuST will assign the initial family member as per stratification and minimisation criteria (see *Randomisation*), with subsequent family member(s) being assigned by TRuST into the same group. All participants will be asked for consent to allow the study team to enquire if another member of their immediate family is enrolled into OPTIMISTIC. Their name will be required to review the randomisation log to establish their study ID. The study ID of the initial family member will be entered into TRuST thereby allowing same group allocation.

### Intervention allocation

Participants will be randomly allocated in a 1:1 ratio* to receive the intervention, or standard patient care. *Except for immediate family member participation (see section *Randomisation*).

### Withdrawal procedures

#### Withdrawal from randomised therapy

Any clinician involved in the usual care of patients may withdraw patients from randomised intervention therapy using his/her clinical judgement. This might occur due to the occurrence of an adverse event and the onset of symptoms that limit tolerability. Participants withdrawn from randomised intervention therapy will remain in the study and will be included in the primary intention-to-treat analysis unless they request that their data are removed. If withdrawal is due to an adverse event it will be logged as such on the Adverse Event Log. All reasons for withdrawal will be noted in the participant’s Case Report Form and medical case notes. The participant and carer will be invited to attend the withdrawal visit. The measurements and activities scheduled at Visit 5 will be performed in the priority set out in the schedule table Appendix incorporating the participants and carers preferences. If a participant is randomised into the study with an immediate family member and either withdraw the other will be able to continue in the study.

#### Withdrawal of consent to follow-up

If at any time the patient formally withdraws his/her consent for future participation and disclosure of future information, no further evaluations will be performed and no additional data should be collected. Data collected before such withdrawal will be retained and used in the study analysis (with consent).

### Temporary suspension of randomised therapy

Temporary discontinuation (no time limit) of randomised therapy may be permitted for justifiable reasons. These periods must be logged accurately. This is important as a secondary analysis will include an ‘on therapy’ versus ‘off therapy’ component using data collected as part of the intervention.

### Participant retention

Due to the complex nature of the condition every effort will be made to ensure participant retention and adherence to the protocol. Patients will be contacted by phone the day prior to each scheduled visit. A study website will also be available for participants to catch-up on study progress (www.optimistic-dm.eu).

### Interventions

#### Behavioural change intervention

In the OPTIMISTIC study patients in the treatment arm will receive cognitive behavioural therapy plus graded exercise therapy. The behavioural change intervention is aimed at increasing the activity level and participation of patients by addressing three core problems thought to maintain disabilities:

1. severe fatigue,
2. a reduced initiative and
3. suboptimal interaction with significant others.

It is assumed that CBT will reduce these problems and thus enable DM1 patients to become more active. The intervention is based on a model of fatigue and disability in DM1 and evidence-based cognitive behavioural interventions for patients with other chronic medical conditions. Intervention group participants will continue to receive standard clinical care as judged necessary by their treating clinicians.

It should be noted that some parts of the intervention are targeted at the caregiver or significant other. If a DM1 patient has no caregiver or significant other, or no caregiver or significant other willing to take part in the study, the patient will NOT be excluded from the study. For brevity, we will use ‘caregiver’ in the following text but this should be taken as meaning ‘caregiver or significant other’.

CBT consists of six different modules. All patients will start with individual goal setting and psycho-education about the role of cognitive-behavioural variables in the disabilities patients’ experience. The patient formulates his or her treatment goals in concrete terms and later on in the therapy the goals are realised step by step by the patient. The treatment is tailored to the patient’s problems: which of the six modules a patient will receive is dependent on the scores on measures that have been collected at baseline assessment. The extra measures for the baseline assessment will take about 40 min. They will be used to assess elements relevant for CBT that will direct the intervention. The extra measures will also enable us to do mediation analysis to determine to what extent the changes in cognitions and behaviour mediate the expected positive effects of the intervention on the outcome measures. If a patient is not able to fill in the questionnaires, it is unlikely that the patient can profit from the intervention and will not be randomised. Based on our previous experience with modular interventions we expect that most patients will receive less than four modules. The six modules are described below:

1. **Learning to compensate for a reduced initiative**. After psycho-education patients learn how to compensate for a reduced initiative by using cues to initiate activity. Examples of these cues are using a diary, mobile phone with an alarm or the scheduling of activities. Caregivers will also be taught how to cue behaviour and how to stimulate the patients to use cues. The goal of this module is to help the patient to start more activities. This module is indicated if a patient scores higher than 38 on Apathy Evaluation Scale (AES). The AES is filled in by the caregiver and/or clinician (or trained assessor) during the intake session. The caregiver is asked to participate in the intervention and to fill in some of the questionnaires. However, if the caregiver refuses or cannot participate, the patient can still take part in the trial.
2. **Suboptimal interaction with caregivers**. The disabilities associated with DM1 put considerable strain on caregivers and can also lead to a negative interaction with the patient. The patients can also feel misunderstood by others, which further hampers their interactions with others. The goal of this module is to optimise the interaction with caregivers by installing realistic expectations about what can be expected from the patient, teach caregivers how to help patients to stay as self-reliant as possible and also reduce caregivers strain by taking time for themselves. The expectations of the patient about the support of others will be discussed and the patient is stimulated to adequately ask for support where possible. This module is indicated if 1) a proxy scores 7 or higher on the Caregiver Strain Index (CSI), 2) the partner and/or patient score 60 or lower on the Marital Satisfaction VAS, or 3) the patient scores 14 or higher on the subscale discrepancy (SSI-D) of the social support inventory (short form).

The following 4 modules are specifically aimed at fatigue maintaining behaviours and beliefs:

3. **Regulation of the sleep-wake pattern (completed for all participants)**. The importance of a regular sleep-wake cycle and good sleep hygiene are discussed, and instructions will be given how to improve both. At baseline patients will register bedtime, get-up times and sleeping during the day for 2 weeks. This will be noted by the patients using a diary. This is plotted in a bar chart to visualise irregularities in the patient’s sleep-wake cycle. This module is indicated if the patient has an irregular sleep-wake cycle and/or scores 60 or higher on the subscale sleep of the Sickness Impact Profile (SIP).
4. **Reformulation of dysfunctional cognitions with respect to fatigue and/or DM1**. At baseline the sense of control over fatigue symptoms, fatigue catastrophising and the tendency to focus on fatigue are assessed. This module is indicated if a patient has a problematic score on one or both of the following instruments: Self-efficacy Scale for fatigue (score 19 or lower), and the Jacobsen Fatigue Catastrophising Scale (score 16 or higher) or a score of 4 or higher on the Illness Management Questionnaire. Helping beliefs with respect to fatigue are formulated and patients practice with them. Patients will also practice with redirecting the focus of attention away from the fatigue toward activity and other sensations. If a patient has dysfunctional beliefs about the illness, for example*,* has difficulty accepting the fact of being ill, helping belief will be formulated. This intervention will be done if a patient scores in the problematic range on the Pictorial Representation of Self and Illness Measure (PRISM), the Beck Depression Inventory Fast Score - (BDI-FS 6 Protocol ≥ 4) or subscale Illness acceptance (≤12).
5. **Activity regulation and graded activity***.* Physical activity will be assessed using an ankle or wrist worn actometer (GeneActiv/Kinesense or equivalent CE marked device). The device is light weight, waterproof and has been adapted for long wear. It is often worn by sports men and women to measure activity progress. Full detailed instructions will be given to the patients at screening/baseline visit. The device will be attached using a non-metal bracelet and patients will be instructed that it should be worn for 14 days continuously but that it can be easily removed by the participant if required by cutting the bracelet with a pair of scissors. The actometer will be worn for 14 days after the screening/baseline assessment to define what activity measures are in place. Relatively active patients first have to distribute their activities more evenly followed by a gradual increase of physical activity. At baseline patients will choose an activity programme with the counsellor, either a low intensity, graded physical activity program and an exercise program aimed at an increased physical fitness:
  - a program aimed at gradually increasing the time that they walk, OR
  - an exercise program aimed at increasing their physical fitness. The exercise programme will be defined through the counselling but will target incorporating moderate intensity exercises such as walking, cycling, jogging or dancing for at least half an hour, three times a week. After participants have increased their physical activity level or fitness they start to increase other activities in order to reach their goals.
    *The Actometer*
    The device will be worn for 14 days continuously after screening or baseline and at months 5, 10 and 16. Additional use may also be suggested by the counselling to assist behaviour change. The device will be fitted at a visit, worn for 14 days, removed and then returned to the trial site by post in a stamped addressed padded envelope. The actometer itself is a tri-axial STMicroelectronics accelerometer and the acceleration will be sampled at 50 Hz. Raw data is transferred through USB to PC and analyses according to patient ID on MOVEeCloud.
    *Non-Wear time*
    Results for a given 14-day period will be deemed invalid if non-wear time exceeds 50 % duration. Where non-wear time is less than 50 % the results will still be used and the non-wear time will be accounted for by imputation of the non-wear time using available wear time data at similar times on other days for the given participant.
    *Control*
    Those not randomised to the intervention will also be invited to wear the actometer for 14 days immediately after visits at screening/baseline, months 5, 10 and 16. The device will be returned by post in the same way as for intervention group participants.
6. **Coping with pain** with a focus on dysfunctional cognitions with respect to pain. Dysfunctional pain cognitions are disputed. More helpful pain-related cognitions will be installed. This module is indicated if a patient has a problematic score on the SF36 pain subscale (score lower than 60) or 44 on the VAS pain.

The intervention will be delivered by therapists who have received extensive training before the trial starts and will use a standardised treatment manual developed by the OPTIMISTIC team. Prior to participating in the graded activity module, the treating clinician will make a judgment as to the degree of physical activity the participant is safely capable of doing, which will be communicated to the therapists. The therapists will remain in contact with the treating clinician throughout the trial to discuss any changes. The therapists and/or the research nurse can request that the treating physician/Investigator to review the patient at any time if any of the research team, the patient or the carer have any concerns. If required, the patients’ GP will be informed.

Participants will receive a workbook containing relevant information and assignments. If deemed appropriate a patient may be given a gym membership to assist with their set goals. If participants give their permission, CBT sessions will be audio recorded and transcribed. Transcriptions will be anonymised. Audio recordings will be stored securely at local sites and destroyed as described under *Study record retention*.

Between sessions participants will do ‘home-work’ assignments that are discussed in the subsequent session. If caregivers are involved in the intervention, they will be asked to support the patient in carrying out these assignments. Treatment integrity will be determined by two experienced cognitive behavioural therapists, who will independently rate a random selection of audio-taped sessions. During the intervention period phone calls to participants will be made at least once a week between sessions to remind them to complete any homework and to ensure attendance at sessions. Where it is felt necessary and resources allow participants will be visited in their homes to increase adherence and provide support where needed.

The intervention runs for 10 months but is front-loaded, meaning the first 4 months can be considered the ‘active’ phase with the remaining 6 months in the ‘booster’ phase. In this period of 10 months a patient will receive 10 to 14 sessions; at least 5 of them are face to face sessions. For the other sessions the therapist can decide, dependent on the traveling distance and the mobility of the patient, to use telephone contact or video conferencing as an alternative.

In addition, all therapists will receive one support call every two weeks by telephone from an experienced cognitive behavioural therapist in the OPTIMISTIC team, with extra support available by email.

#### Comparison

Standard care is the usual care at the participating site. This will vary by site and according to individual patient symptoms but the minimum is generally an annual visit plus ECG/EKG. Other care may include appropriate history taking, physical examination, ECG/EKG (or more in depth cardiac investigations), pulmonary and gastrointestinal investigations, and rehabilitation measures.

All control group participants will visit their trial site at baseline and months 5, 10 and 16 months post intervention (that is, 16 months from baseline) for the measurements as detailed in Appendix.

### Study and safety assessments

A number of factors affecting the trial population suggest that we would expect to observe a larger than normal incidence of episodes of adverse events (AEs) such as fatigue and ill health due to co-morbidities of the study population. All known disease progression and co-morbidities will be noted but not reported.

Due to the large amount of co-morbid diseases that we expect to be present in the population, we will record, but not report, Serious Adverse Events (SAE) in the following categories:

1. Any new cardiovascular event,
2. Any new treatment of myotonic dystrophy type 1,
3. Any hospitalisation due to falls or fractures,
4. Any hospitalisation due to exacerbation of an existing medical condition, and
5. Any elective or planned investigation or treatment.

All unexpected SAEs (that is, an SAE that is not listed above) will be recorded and reported to the sponsor. An example of an unexpected SAE would be premature death of a participant that was not due to an existing condition.

Safety assessments will be recorded at every visit.

### Data collection and management

#### Data collection

It is the responsibility of the local PI to ensure the accuracy of all data entered and recorded in the CRFs and the database for his or her site. The Delegation of Responsibilities Log will identify all trial personnel responsible for data collection, data entry and handling and managing the database.

The data will be collected by the PI or delegate onto a paper CRF for subsequent transcription to the eCRF. Data entry onto the web based eCRF may be done locally or paper CRFs may be sent to an external unit for data entry depending on local preference. If CRFs are sent away for data entry a copy of what is sent must be retained by the originating team. This includes patient-completed questionnaires. The two copies must always record the same information. This means that if values are updated as a result of the querying process the changes must be annotated on both copies, initialled and dated. Both physical and electronic storage of data will protect the security of these data and the confidentiality of participants.

The study questionnaires will be completed according to the schedule shown in Appendix by the participant with the assistance of the research nurse and caregivers. Laboratory derived blood tests will be held on local clinical databases in an identifiable format and for a time frame in line with local regulations and policies for clinical care systems. All research blood samples will be processed and stored at each site (as per Sample Analysis and Chain of Custody Plans in the Study Operations Manual) and transported to the relevant laboratory for analysis at timeframes outlined in the Study Operations Manual for batch analysis.

The medical notes can act as source data for past medical history, subsequent medical conditions, hospital admissions, diagnostic reports and blood and urine results.

#### Data management system

A data management system will be provided by TCTU using OpenClinica (https://www.openclinica.com/), its standard Good Clinical Practice (GCP)-compliant data management system. The study system will be based on the protocol and CRF for the study and individual requirements of the investigators. Development and validation of the study database, quality control and extraction of data will be done according to TCTU procedures.

### Statistics and data analysis

#### Sample size calculation

The primary outcome is the DM1-Activ scale, which is a Rasch-built measure of activity and participation for patients with DM1 and is currently the best such measure for these patients. Based on a minimum clinically important mean difference of 1.4 on the 40-item DM1-Activ scale, standard deviation of 3.5 and effect size = 0.4, 80 % power and 5 % significance level, we need a sample size of 100 in each arm, or 200 in total.

DM1 is a genetic condition so it is likely that some members of the same family will take part in the study. To avoid the risk of contamination, immediate family members (mother, father, children, and siblings) will be allocated to the same group, with the first family member being randomly allocated and subsequent members being assigned to the same group as the first. This means that there will be some clusters of more than one individual and the sample size needs to be inflated to account for this. Newcastle estimates that this may affect 50 % of their participants, the other sites estimate that fewer than 10 % of participants will be part of a family group. All sites estimate that almost all family groups will comprise two individuals. Using estimates of distributions of recruitment across sites (Newcastle = 23 %; Nijmegen = 30 %; Paris = 33 %; and Munich = 13 %) and the above family group proportions gives an average cluster size of 1.17. Using a conservative intra-cluster correlation coefficient of 0.20, gives an inflation factor of 1.035, meaning the recruitment target needs to be increased by 10 to 296. After allowing for up to 30 % drop-out this gives a final sample size of 208, or 104 participants per arm.

These are conservative estimates (especially the minimum clinically important difference). The clinical OPTIMISTIC sites between them see well over 1,000 DM1 patients per year, which gives OPTIMISTIC a good pool of potentially eligible participants. Around 70 % of DM1 patients are expected to be eligible for OPTIMISTIC.

We will be using one of our secondary outcomes, the 6-minute walk test (6MWT) as an additional validation of the DM1-Activ scale, meaning that we have also powered this secondary outcome. The DM1-Activ sample size of 208 gives over 90 % power for the 6MWT based on a mean difference of 40 m, standard deviation of 80 m and a baseline of 450 m.

#### Proposed analysis

The primary analyses will be conducted according to the principles of intention to treat (ITT) as outlined on the ICH E9 ‘Statistical Principles for Clinical Trials’.

Continuous variables will be summarised by the number of observations, number of missing values, mean, standard deviation (SD), median, inter-quartile range (IQR) and range. Summaries will be provided at baseline, at each subsequent time point and for the change from baseline by intervention group. Categorical variables will be summarised by the number of observations, number of missing values and number and percentage in each category. Summaries will be provided at baseline and at each subsequent time point.

The primary outcome and most other outcomes are continuous and so linear models will be utilised. The primary outcome of change in DM1-Activ will be analysed using mixed effects (repeated measures) regression models. Models will include fixed effects for intervention group, time point, and their interaction, plus random effects for each subject to account for repeated measures, and will assume a general covariance structure. A binary variable will be added to the regression model to represent the difference between the intervention and standard care. The minimisation variables of severity of DM1 (measured using the MIRS 5-point scale) and caregiver involvement at baseline will also be added to the regression model as fixed effects, as well as stratification by site.

Intervention effect differences will be reported with 95 % confidence intervals (CIs) and *P*-values. Results will also be explored and reported with additional *a priori* adjustment for potential effect modifiers (see list in *Measures used as potential effect modifiers*). Subgroup analyses will be carried out by first testing for a subgroup factor by intervention interaction [16]. If this is significant at the 5 % level, results will be estimated separately by the different subgroups. These analyses will also be repeated for all the secondary outcomes. Appropriate transformations of outcomes will be performed where necessary to satisfy modelling assumptions.

A statistical analysis plan (SAP) will be written prior to datalock and approved by the statistician and CI. Analyses will be carried out using SAS.

#### Missing data

The extent of missing data will be explored in the outcomes especially the primary outcome. Patterns of missing data will be explored and predictors of missingness examined, especially if these vary by intervention. If necessary, multiple imputations will be utilised to impute missing data assuming the missingness mechanism is missing at random (MAR). However, mixed models have the useful property of using all available data without resorting to imputation where data is MAR. The assumption of MAR cannot be pre-specified or tested as it depends on whether and how data has become missing. Measures should be taken where possible to minimise the extent of missing data in the recording of outcomes so that missing data is less likely.

### Transfer of data

Case Report Forms (CRF) will be developed together with the trial management team, statistician and data manager to ensure that the data management system (see *Data management system*) supports the research aims of the study. The data management system will be fully validated, including the provision of test data and supporting documentation. Data entry will be coordinated by TCTU although sites will generally be doing their own data entry. It is the PI responsibility to ensure the accuracy of all data entered and recorded in the CRF/eCRFs. Data will be stored on servers controlled by TCTU and housed at the University of Dundee. Backup-up and disaster recovery will be provided by TCTU according to its standard operating procedures.

The study questionnaires will be completed at screening/baseline, 5-month, 10-month (that is, the end of the intervention) and 6-month post-intervention onto a paper CRF with subsequent transcription (where appropriate) to OpenClinica.

All research related blood sample analysis and data established from the NHS/health care tests will be stored in an unidentifiable format in the password protected disaster recovery formatted database.

All research blood samples (link-anonymised) will be processed as per Sample Analysis and Chain of Custody Plans in the Study Operations Manual and transported to the relevant laboratories for analysis.

Patients will be informed of data storage and consent will be sought. Finally, the Delegation of Responsibilities Log will identify all trial personnel responsible for data collection, entry, handling and managing the database.

### Trial management and oversight arrangements

OPTIMISTIC will have the following two oversight committees:

1. The Trial Management Committee will be responsible for day-to-day operational issues.
2. The External Advisory Board will be responsible for strategic decision making and issues linked to monitoring ethical and patient safety issues, together with monitoring recruitment and data quality issues.

#### Trial management committee

The OPTIMISTIC trial will be managed by TCTU. The Trial Management Committee (TMC) will comprise the leader of the trial component of OPTIMISTIC, senior statistician, TCTU Assistant Director, a representative of TCTU’s data management staff and the TCTU senior trial manager and study trial manager. Other members of the OPTIMISTIC group may be added as required by the consortium or the Trials Unit. The TMC will meet at least monthly by face-to-face and more often if needed, especially in the early phases of the trial.

#### Trial/study management

Each clinical site will have a research nurse or research associate allocated to the study who will oversee the study and will be accountable to the local PI. The research nurse or research associate will be responsible for checking the CRFs for completeness, plausibility and consistency. Any queries will be resolved by discussion with the CI, or delegated member of the study team. At every site a lead CB therapist will be responsible for the referral of patients to an appropriate CB therapist if they are randomised to the intervention arm. The same CB therapist will have regular meetings with the other therapists at the site and will oversee the flow of patients in the intervention arm. The CB therapist will be responsible for data collection at the clinical site with respect to the intervention.

A study-specific Delegation Log will be prepared for each site, detailing the responsibilities of each member of staff working on the study.

#### External advisory board

The External Advisory Board comprises independent, international experts covering the key areas of competence required to provide oversight of OPTIMISTIC scientific activity. The list of participants is at http://optimistic-dm.eu/external-advisory-board/. The Board ensures a high standard of research and governance and monitors the progress of the project. The OPTIMISTIC consortium will consult the Board at least annually (more often if the Board requires) to get members’ opinions with regard to study progress, especially on issues linked to the trial.

The Board will comment on ethical and safety issues as well as general conduct issues. In addition the project will have access to the TREAT-NMD project ethics council (PEC) if any additional advice should need to be sought.

#### Inspection of records

The CI, PIs and all institutions involved in the trial will permit trial-related monitoring, audits, and ethical review. The CI agrees to allow each country specific sponsor or, representatives of the sponsor, direct access to all trial records and source documentation.

### Good clinical practice

#### Ethical conduct of the study

The study will be conducted in accordance with the principles of Good Clinical Practice (GCP). In addition to sponsorship approvals, a favourable ethical opinion will be obtained from the appropriate country specific ethics committee(s) and where appropriate local management approval(s) will also be obtained prior to the start of the trial.

#### Confidentiality

All laboratory specimens, evaluation forms, reports, and other records will be identified in a manner designed to maintain participant confidentiality. All records will be kept in a secure storage area with limited access by appropriate trial staff only. Clinical information will not be released without the written permission of the participant, except as necessary for monitoring and auditing by the sponsors or their designee. The CI and staff involved with the trial will not disclose or use for any purpose other than performance of the trial, any data, record, or other unpublished, confidential information disclosed to those individuals for the purpose of the trial. Prior written agreement from the sponsors or their designee will be obtained for the disclosure of any said confidential information to other parties.

#### Data protection

The CI and staff involved with the trial will comply with the requirements of the relevant data protection legislation for their jurisdiction with regard to the collection, storage, processing and disclosure of personal information. The CI and trial staff will also adhere, if appropriate, to other local regulations and policies with regard to confidentiality of data. Access to collated participant data will be restricted to the CI and appropriate trial staff.

Computers and IT systems used in connection with the trial will have access-control measures such as centrally allocated logins and passwords. Finally, published results will not contain any personal data that could allow identification of individual participants.

#### Insurance and indemnity

##### Insurance

Each country-specific sponsor will obtain and hold a policy of public liability insurance for legal liabilities arising from the study at each of its identified sites. The site ISF and TMF will hold notification of sponsor and a copy of certificate of insurance as per local sponsor policy.

##### Indemnity

The country specific sponsor will provide study participants with indemnity in relation to participation in the study but has insurance for legal liability as described above.

#### Study conduct responsibilities

##### Protocol amendments, deviations and breaches

The CI will seek approval for any amendments to the protocol or other study documents from the sponsor and relevant ethics and local management committees. Amendments to the protocol or other study documents will not be implemented prior to these approvals. In the event that a PI needs to deviate from the protocol, the nature of and reasons for the deviation will be recorded in the CRF, documented and submitted to the sponsor. If this necessitates a subsequent protocol amendment, this will be submitted to the sponsor for approval and then to the appropriate ethics and local management committees for review and approval.

In the event that a serious breach of GCP is suspected, this will be reported to the site-specific sponsor immediately.

#### Study record retention

The University of Dundee will archive OpenClinica electronic data and any other trial materials for five years after trial closure. Participating sites will store their trial materials locally according to local archiving regulations but for not less than five years.

#### End of trial

The end of trial is defined as database lock. The sponsor, CI and/or the external advisory board have the right at any time to terminate the study for clinical or administrative reasons. The end of the trial will be reported to the sponsor and ethics committee(s) within 90 days, or 15 days if the trial is terminated prematurely. The CI will ensure that any appropriate follow up is arranged for all participants. A summary report of the trial will be provided to the sponsor and ethics committee(s) (in English) within 1 year of the end of the trial.

#### Reporting, publications and notification of results

##### Authorship policy

Ownership of the data arising from this study resides with the trial team and their respective employers. Authorship of publications coming from this work will be in accordance with OPTIMISTIC’s publication policy.

##### Peer review

The OPTIMISTIC proposal, including this trial, was reviewed by external reviewers appointed by the European Commission as part of the 7th Framework funding review process.

#### Ethical approval

Version 3.2 of the protocol has received ethical approval from the National Research Ethics Service Committee North East - Sunderland (UK), Comite de Protection des Personnes ile de France V (France); Ethikkommission bei der LMU München (Germany); Concernstaf Kwaliteit en Veiligheid Commissie Mensgebonden Onderzoek Regio Arnhem- Nijmegen (Netherlands).

## Discussion

DM1 is one of the most variable human diseases, and has complex, multi-systemic and progressively worsening clinical manifestations. It leads to severe physical impairment, restricted social participation and premature death. The complexity of DM1 poses a challenge for patient management and treatment options are currently extremely limited. There is a strong need to improve clinical practice in the management of these patients suffering from a rather neglected disease.

OPTIMISTIC is the first model-based clinical trial in DM1 and it will provide a unique set of data. The project will investigate the serious gaps in our knowledge of the natural history of DM1, patient-relevant outcome measures, and evaluate the effectiveness of CBT and physical exercise on quality of life in a trial designed to be highly applicable to other contexts and jurisdictions. Furthermore, OPTIMISTIC will advance our knowledge of individual and composite biomarkers of the disease. Finally, the project will fully explore scientific questions about moderating and/or mediating factors of the short- and long-term clinical response, together with the short- and long-term safety of CBT and physical activity. This body of work will not only improve our understanding of the relevant determinants of the prognosis of DM1 but will also produce effectiveness data on an intervention that could fill a treatment-gap for DM1 patients.

The OPTIMISTIC study started on 1/11/2012 and will end 31/10/2016. Recruitment started on 6th April 2014.

## Trial status

Ongoing and recruiting at time of submission.

## Appendix

**Table 1:** Trial outcome measurement schedule

			Measurement point				
Priority	outcome measure	Time to complete	Visit 1 Screening	Visit 2 Baseline ^a^	Visit 3 (5 months)	Visit 4 (10 months)	Visit 5 (16 months)
1	Demographics		X				
	General medical history		X	X			
1	Concomitant medications & therapies		X	X	X	X	X
	Adverse events			X	X	X	X
1	Confirm genetically proven classical or adult DM1/specific DM1 history		X	X			
	Primary outcome measure						
1	DM1-Activ	5 min		X	X	X	X
	Secondary outcome measures						
	Activity						
5	MDHI	20 min		X	X	X	X
2	6-minute walk test/BORG Scale	10 min	X	X^a^	X	X	X
13	Actometer data	For 14-days after visit	X	X^a^	X	X	X
	Fatigue and sleepiness						
3	Checklist individual strength (CIS) fatigue	5 min	X	X^a^	X	X	X
11	Fatigue and daytime sleepiness scale (FDSS)	5 min		X	X	X	X
	Quality of Life						
6	Individualised neuromuscular quality of life (InQoL)	20 min		X	X	X	X
	Mood						
4	Beck depression inventory-fast screen (BDI-FS):	10 min	X	X^a^	X	X	X
	Cognitive Measures						
8	Apathy evaluation scale	10 min		X	X	X	X
12	Stroop	5 min		X	X	X	X
	Potential Effect Modifiers						
9	MIRS	2 min	X	X^a^	X	X	X
14	McGill pain questionnaire	10 min		X	X	X	X
15	Trail making	10 min		X	X	X	X
21	Adult social behaviour questionnaire (ASBQ)	10 min		X	X	X	X
	Marital satisfaction (VAS)			X	X	X	X
16	CBT: self-efficacy scale for fatigue (SES-28);	4 min		X	X	X	
17	CBT: fatigue catastrophising scale (FCS)	4 min		X	X	X	
18	CBT: focusing on symptoms (IMmQ)	4 min		X	X	X	
19	CBT: Illness acceptance scale	4 min		X	X	X	
20	CBT: social support (SSL-D)/Social Support list interactions (SSL-I)/Social support list negative interactions (SSL-N)	4 min		X	X	X	
	Sickness impact profile (subscale sleep)			X			
	Biomarkers						
7	Whole blood 10 ml sample x3 (10 ml for DNA; 10 ml for RNA and 10 ml serum).	5 min		X		X	X
22	Urine sample (10 ml or more) x1	5 min		X		X	X
	Carers measures						
	Caregivers strain index (CSI)			X	X	X	X
	ASBQ (Other)	10min		X	X	X	X
	Marital satisfaction (VAS) (can be a partner).			X	X	X	X
	Will not be performed if carer is a non-partner.						
	Apathy evaluation scale (AES)			X	X	X	X

^a^Visit 1 and Visit 2 may be combined if appropriate. No duplication of tests/questionnaires performed

## Abbreviations

ADL: activities of daily living
AE: adverse event
AES: apathy evaluation scale
ASBQ: adult social behaviour questionnaire
ATP: adenosine triphosphate
BDI-FS: Beck depression inventory fast screen
BMI: body mass index
CBT: cognitive behavioural therapy
CI: chief investigator
CIS-fatigue: checklist individual strength fatigue subscale
CMRI: cardiac magnetic resonance image
CRF: case report form
CTG: cytosine, thymine, guanine
CTIMP: clinical trial of an investigational medicinal product
DM1: myotonic dystrophy type 1
DMPK: dystrophia myotonica-protein kinase
DNA: deoxyribonucleic acid
ECG/EKG: electrocardiography
FCS: fatigue catastrophising scale
FDSS: fatigue and daytime sleepiness scale
FSHD: facioscapulohumeral muscular dystrophy
GCP: good clinical practice
ICH: International Conference on Harmonisation
IMmQ: illness management questionnaire
InQoL: individualised neuromuscular quality of life questionnaire
IQR: inter-quartile range
ISF: investigator site file
MAR: missing at random
MDHI: myotonic dystrophy health index
MIRS: muscular impairment rating scale
MR: magnetic resonance
MRI: magnetic resonance imaging
mRNA: messenger ribonucleic acid
PCr: polymerase chain reaction
PI: principal investigator
PIS: participant information sheet
PT: physiotherapy
QQ: questionnaire
RF: radiofrequency
RNA: ribonucleic acid
RyR: ryanodine receptor
SAE: serious adverse event
SAP: statistical analysis plan
SD: standard deviation
SERCA: sarco/endoplasmic reticulum Ca2+-ATPase
SES: self-efficacy scale
SIP: sickness impact profile
SSL-D: social support list discrepancy
SSL-I: social support list interactions
SSL-N: social support list negative Interactions
TIRM: turbo inversion recovery magnitude
TCTU: Tayside Clinical Trials Unit
TRuST: Tayside randomisation system
VAS: visual analogue scale
6MWT: 6-minute walk test
